# CT Imaging Features of Pulmonary Sarcoidosis: Typical and Atypical Radiological Features and Their Differential Diagnosis

**DOI:** 10.3390/medicina61122094

**Published:** 2025-11-25

**Authors:** Elisa Baratella, Valeria di Luca, Alessandra Oliva, Ilaria Fiorese, Antonio Segalotti, Marina Troian, Stefano Lovadina, Barbara Ruaro, Francesco Salton, Roberta Polverosi, Maria Assunta Cova

**Affiliations:** 1 Radiology Unit, Department of Medical Surgical and Health Sciences, University Hospital of Cattinara, 34149 Trieste, Italyantoniosegalotti@gmail.com (A.S.);; 2 Radiology Unit, Cattinara University Hospital, 34149 Trieste, Italy; fiorese.ilaria@gmail.com; 3 Thoracic Surgery Unit, Cattinara University Hospital, 34149 Trieste, Italy; 4 Pulmonology Unit, Department of Medical Surgical and Health Sciences, University of Trieste, 34149 Trieste, Italy; 5 Independent Researcher, 35123 Padua, Italy

**Keywords:** pulmonary sarcoidosis, high-resolution computed tomography (HRCT), granulomatous lung disease, differential diagnosis, imaging features, radiological patterns

## Abstract

Sarcoidosis is a chronic, idiopathic, multisystemic inflammatory disease characterized by non-caseating granulomas, most commonly affecting the lungs and mediastinal lymph nodes. Radiological imaging plays a fundamental role in the diagnosis, assessment of disease extent, and differentiation from other pulmonary conditions. This narrative review offers a comprehensive overview of the imaging features of pulmonary sarcoidosis, focusing on both typical patterns—such as bilateral hilar lymphadenopathy, perilymphatic nodules, and upper lobe-predominant infiltrates—and atypical manifestations—including alveolar opacities, miliary nodules, fibrocystic changes, and lower lobe involvement. Emphasis is placed on the utility of high-resolution computed tomography (HRCT) in detecting early parenchymal changes and complications such as fibrosis, bronchiectasis, and pulmonary hypertension. Differential diagnosis, including tuberculosis, silicosis, metastatic disease, organizing pneumonia, and hypersensitivity pneumonitis, are discussed to aid interpretation. Recognizing the spectrum of radiological presentations is essential for distinguishing sarcoidosis from other interstitial and granulomatous lung diseases. Radiologists play a pivotal role in the multidisciplinary diagnostic process, contributing to timely diagnosis, risk stratification, and optimized patient management.

## 1. Introduction

Granulomatous lung diseases (GLDs) represent a heterogeneous group of pulmonary disorders that can be broadly classified into infectious and non-infectious etiologies. This distinction is clinically significant, as misdiagnosis can lead to inappropriate treatment strategies with potentially severe consequences for the patient [1]. Among the principal non-infectious GLDs, sarcoidosis stands out as a chronic, idiopathic, multisystemic inflammatory disease characterized by the formation of non-caseating granulomas [2].

Histologically, these granulomas are composed of organized aggregates of inflammatory cells, including activated macrophages (epithelioid histiocytes), Langhans-type multinucleated giant cells [3], and surrounding lymphocytes arranged in a palisading pattern, often accompanied by plasma cells and peripheral fibroblasts. Giant cells may contain characteristic—but non-specific—cytoplasmic inclusions such as asteroid (star-shaped) bodies, Schaumann bodies (i.e., concentric calcifications), and Hamazaki-Wesenberg bodies (i.e., lipofuscin-like inclusions resembling fungal spores). Although typically non-necrotizing, granulomas in sarcoidosis may exhibit focal necrosis in up to 14% of cases [4].

**Figure 1:**
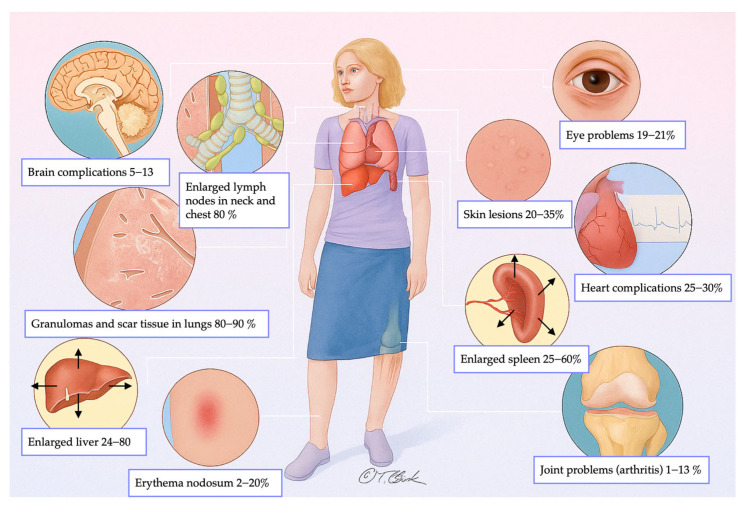
Frequency distribution of multi-organ involvement in sarcoidosis.

Sarcoidosis can affect virtually any organ system. The most frequently involved sites include skin (20–35%), eyes (19–21%), liver (24–80%), spleen (25–60%), heart (25–30%), joints (1–13%), nervous system (5–13%), and musculoskeletal system (1–13%). Lymphadenopathy—both thoracic and extra-thoracic—is present in approximately 80% of patients [1,5]. However, pulmonary involvement and enlargement of mediastinal and hilar lymph nodes are the most common manifestations, observed in up to 90% of cases [6]. Notably, around 50% of these patients remain asymptomatic, and abnormalities are often detected incidentally in imaging studies [7,8]. Figure 1 illustrates the frequency of multi-organ involvement in sarcoidosis.

## 2. Etiology and Diagnosis

The exact etiology of sarcoidosis remains largely unknown, though it is widely believed to result from a complex interaction between genetic susceptibility and environmental antigenic exposure. This interaction likely influences both the risk of developing the disease and the wide range of clinical phenotypes observed. The triggering antigen may vary according to ethnicity, geographic location, and individual genetic background [9,10,11]. For instance, the incidence is lower in Japan compared to Northern European countries, and in the United States it is higher among African Americans (36 cases per 100,000 people) than in the Caucasian population (11 cases per 100,000 people) [5,12,13]. The heterogeneity of sarcoidosis is not limited to its incidence but extends to its clinical presentation. In at least 50% of cases, spontaneous resolution occurs without persistent damage. However, in a minority of cases, significant permanent impairment of major organs may lead to a marked reduction in life expectancy [9,14,15].

### 2.1. Diagnostic Criteria and Imaging Modalities

Diagnosis requires the integration of clinical, radiological, and histopathological data, and it is typically established by meeting the following criteria: (1) Presence of clinical and/or radiological abnormalities consistent with the disease; (2) Histological confirmation of non-caseating granulomas; and (3) Exclusion of other conditions with similar histological and clinical features [3,16].

Traditionally, chest radiography (CXR) has been the first imaging modality used to evaluate pulmonary involvement and formed the basis of the Siltzbach classification, which categorizes pulmonary sarcoidosis into five stages (Table 1) [17]. However, CXR lacks sensitivity for early disease and provides limited information on disease activity or extent [2,6,7]. High-resolution computed tomography (HRCT) offers superior sensitivity and spatial resolution, enabling the detection of subtle parenchymal abnormalities and early interstitial involvement, therefore largely supplanting CXR for detailed assessment, especially in early or atypical cases [17,18].

The HRCT scan must be acquired using a dedicated technical protocol (volumetric acquisition, thickness less than 1.5 mm, high spatial resolution reconstruction, shortest rotation time highest pitch) with the patient in a supine position, during a single breathe hold at full inspiration. An expiratory acquisition could be obtained at end-expiration to evaluate the presence of air trapping [19].

While CXR and CT remain the initial imaging modalities for evaluating suspected or confirmed pulmonary sarcoidosis [17,18], additional techniques may be used in specific clinical scenarios [20]. In advanced stages of pulmonary sarcoidosis, particularly when fibrotic changes are present, assessing disease activity becomes challenging. Distinguishing between potentially reversible granulomatous inflammation and irreversible fibrosis is critical for guiding treatment decisions [21,22,23,24]. In this context, integrated positron-emission tomography with fluorodeoxyglucose (18-FDG PET/CT) can help identify metabolically active disease, enabling assessment of inflammatory activity and detection of potentially occult extra-pulmonary involvement [25,26,27]. Moreover, this modality may also aid in monitoring therapeutic response, as reductions in FDG uptake often correlate with clinical improvement [21,24].

PET/CT also plays a key role in the diagnosis and follow-up of cardiac sarcoidosis, with reported sensitivity and specificity of 89% and 78%, respectively [28,29]. Cardiac magnetic resonance imaging (MRI) serves as another important diagnostic tool, demonstrating even higher sensitivity (95%) and specificity (85%) [30]. However, MRI has limited utility in assessing pulmonary involvement due to its lower spatial resolution, reduced signal-to-noise ratio, and susceptibility to artifacts arising from air-tissue interfaces and respiratory or cardiac motion [31].

### 2.2. Histopathologic Confirmation

Although radiologic findings may strongly suggest sarcoidosis, histological confirmation remains essential. Historically, the Kveim skin test was used for diagnosing sarcoidosis through the intradermal injection of a sarcoidosis extract. However, due to concerns about its reliability and lack of standardization, the test has largely been replaced by more modern diagnostic techniques [32]. Nowadays, tissue samples are typically obtained via bronchoalveolar lavage (BAL) and/or transbronchial lymph node biopsy.

BAL is particularly useful for collecting biological material, but its diagnostic yield can be limited. Endobronchial ultrasound-guided transbronchial needle aspiration (EBUS-TBNA) improves accuracy by targeting hilar or mediastinal lymph nodes. If these approaches are inconclusive, surgical biopsy may be required to obtain a larger, more representative sample for histopathological analysis [33,34,35].

### 2.3. The Role of Pulmonary Function Tests

Pulmonary function tests (PFTs) may have a role at multiple levels in sarcoidosis management, offering insight into disease severity, functional impairment, and therapeutic response [20]. Although many patients maintain normal pulmonary function, 10–30% may experience a reduction in lung capacity, often indicative of more severe or chronic disease forms [36]. Early functional impairment often correlates with a poorer long-term prognosis [20].

A ≥5% decrease in forced vital capacity (FVC) has been suggested to be associated with disease progression, while an amelioration can correlate with a response to treatment [37,38,39].

Additional metrics, including forced expiratory volume in one second (FEV1) and FEV1/FVC ratio, are important in evaluating airway obstruction, particularly when bronchial distortion, peripheral lymph node compression, or endobronchial involvement are present [40,41]. Up to 20% of sarcoidosis patients develop pulmonary fibrosis [42], which typically results in decreased diffusing capacity of the lungs for carbon monoxide (DLCO) [42,43].

CT scans showing fibrosis involving more than 20% of lung parenchyma are associated with worse outcomes [44,45]. Regular monitoring of FVC and DLCO is crucial for detecting disease progression, as a ≥10% decline in DLCO or ≥5% reduction in FVC from baseline should prompt closer clinical evaluation and may necessitate treatment escalation [20]. A disproportionate drop in DLCO, especially when accompanied by persistent dyspnea, may also indicate emerging pulmonary hypertension [46,47,48,49].

The six-minute walk test (6MWT) is a simple and reproducible method for evaluating functional capacity in patients with sarcoidosis and offers valuable prognostic insights. A walking distance of less than 300 m is linked to an increased risk of disease progression and reduced survival, whereas a significant decline in peripheral oxygen saturation (≥4% from baseline to the end of exercise) reflects greater disease severity and poorer exercise tolerance, and may warrant further investigations, such as assessment for pulmonary hypertension [50].

## 3. Lymphadenopathy

### 3.1. Typical Imaging

**Figure 2:**
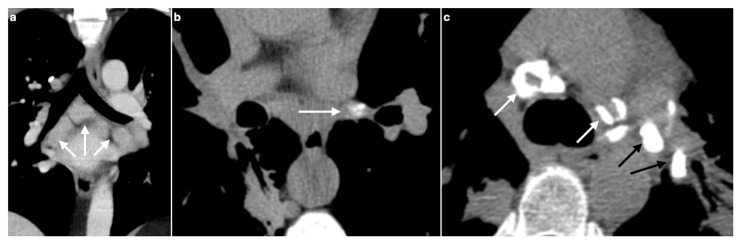
(**a**) MPR coronal plane shows mediastinal lymph node enlargement, bilateral and symmetric (white arrows). (**b**) Lymph nodal calcifications may appear as punctate (white arrows), (**c**) egg-shell (white arrows) or dense (black arrows).

The most characteristic radiological finding in sarcoidosis is bilateral and symmetric hilar lymphadenopathy, often accompanied by mediastinal lymph node enlargement (Figure 2a). This pattern is observed in approximately 95% of patients [7,51,52]. Thoracic lymphadenopathy is typically bilateral, with a right-sided predominance [9]. When bilateral hilar lymphadenopathy is seen in conjunction with right para-tracheal lymph node enlargement, the resulting pattern—known as the Garland’s triad (or “lambda sign”, when seen on gallium scintigraphy)—is considered a classic radiological feature of sarcoidosis [53,54].

#### Calcifications

Lymphadenopathy in sarcoidosis may occasionally exhibit calcifications, a feature also seen in other chronic granulomatous diseases. The presence of nodal calcifications is typically associated with longer disease duration [2]. At the time of diagnosis, calcifications are observed in approximately 20% of cases, increasing to nearly 50% in patients with long-standing sarcoidosis [55]. These calcifications may present in various forms, including amorphous, punctate (Figure 2b), “popcorn-shaped”, or “egg-shell” patterns (Figure 2c) [2].

### 3.2. Atypical Imaging

Unilateral hilar lymphadenopathy is observed in less than 5% of patients with atypical sarcoidosis, more commonly affecting the right hilar region than the left [56]. This finding may occur in isolation or in combination with right paratracheal lymphadenopathy. Less commonly, lymph node involvement may be limited to mediastinal, paratracheal, or subaortic regions, without associated hilar lymphadenopathy [56,57,58,59].

## 4. Pulmonary Interstitium

### 4.1. Typical Imaging

From an imaging perspective, parenchymal involvement is observed in nearly half of sarcoidosis patients and is marked by significant morphological variability [9]. The most characteristic pattern is the presence of multiple granulomas appearing as micronodules with a perilymphatic distribution, seen in approximately 75–90% of cases. These nodules are typically small (2–4 mm in diameter), round, well-defined, and usually bilateral, with a frequently—but not universally—symmetrical distribution. They predominantly affect the upper and middle lobes [17].

**Figure 3:**
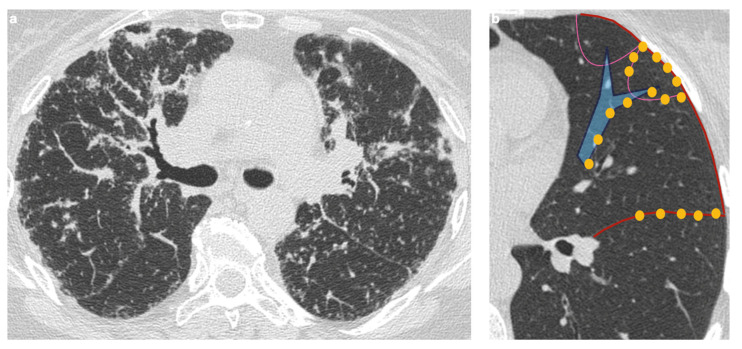
(**a**) High-resolution CT scan in the axial plane shows the presence of diffuse micronodules with a peri lymphatic distribution. (**b**) Those well-defined micronodules (yellow points) are typically seen along bronchi (blue lines), interlobular septa (pink lines), fissures and pleura (red line).

Owing to the perilymphatic distribution of sarcoid granulomas, HRCT typically reveals numerous micronodules located along the peribronchovascular interstitium, interlobar fissures, and interlobular septa (Figure 3) [54].

Over time, these granulomas may coalesce to form larger lesions (macronodules), which can distort the normal lung architecture [57,60,61]. The key imaging features of sarcoid granulomas are summarized in Table 2.

### 4.2. Atypical Imaging

#### 4.2.1. Nodules and Masses

A distinguishing feature of atypical sarcoidosis on HRCT is the presence of nodules or masses measuring 1–4 cm, which can result from the coalescence of multiple granulomas. These lesions may be multiple, bilateral, and are typically located in the subpleural or perihilar regions [62]. In some cases, smaller nodules are seen surrounding these larger masses, predominantly in a peripheral distribution—an appearance known as the “galaxy sign” [63].

**Figure 4:**
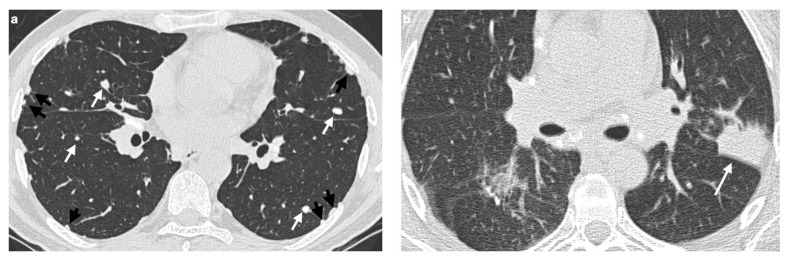
(**a**) Chest CT scan demonstrates the presence of parenchymal nodules (white arrows) and multiple nodules in the subpleural areas of both lungs (black arrows). (**b**) in a patient with known sarcoidosis, it is possible to recognize an area of consolidation in upper left lobe (white arrow); bilateral lymphnodal calcification in the mediastinum can also be noted.

Another recently described imaging feature is the “sarcoid cluster” sign, characterized by the presence of numerous micronodules aligned along the interlobular septa, most often in the supleural areas and particularly in the peripheral regions of the upper and middle lobes [64] (Figure 4a). In rare instances, these micronodules may merge into larger masses, extending into the peribronchovascular interstitium surrounding bronchi and perihilar vessels [2]. Histopathologically, the “sarcoid cluster” corresponds to multiple non-caseating, non-coalescent granulomas composed primarily of CD4+ T-lymphocytes, in the absence of fibrosis [56,57,60,61,65].

#### 4.2.2. Patchy Parenchymal Consolidation

On HRCT, patchy parenchymal consolidations represent areas of lung opacification caused by the coalescence of micronodules that infiltrate or compress the alveoli. These consolidations are typically located in the peribronchovascular regions, with a predilection for the upper and middle lobes (Figure 4b). They are usually bilateral and symmetric in distribution and occur in approximately 10–20% of patients with sarcoidosis [2,58,66].

#### 4.2.3. Ground-Glass Opacities

Ground-glass opacities (GGOs) are observed in approximately 40% of patients with pulmonary sarcoidosis and are unevenly distributed throughout the lung parenchyma [2]. These opacities are generally caused by the merging of micronodules and interstitial fibrotic changes, leading to compression of the airways without actual filling of the alveolar spaces [7,67].

Typically, GGOs present with indistinct margins. However, in rare cases where intra-alveolar granulomas, desquamative cells, or hyaline membranes are present, the margins may appear well-defined [2]. A distinct form of GGO surrounded by a nearly complete ring of parenchymal consolidation is known as the “inverted halo sign” or “atoll sign”—a radiologic finding described in sarcoidosis as well as in other granulomatous diseases [68,69].

#### 4.2.4. Miliary Opacities

The military pattern is a rare radiologic manifestation of pulmonary sarcoidosis, occurring in fewer than 1% of cases. When this pattern is identified, it is essential to consider alternative, more common causes with similar imaging features—such as tuberculosis, pneumoconiosis, or pulmonary metastases—before attributing it to sarcoidosis [70].

## 5. Fibrosis

**Figure 5:**
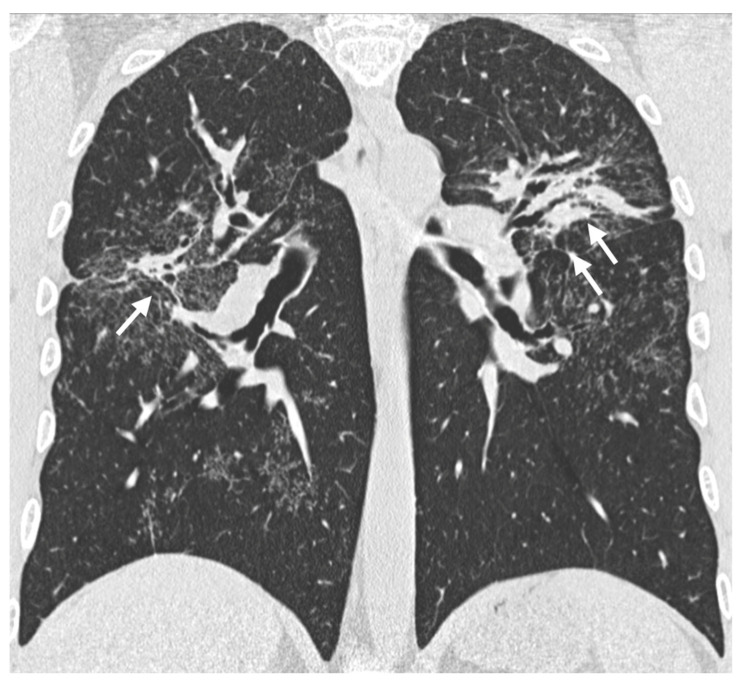
MPR reconstruction on coronal plane clearly shows linear bands of fibrosis (white arrows) typically radiate away from hila in all directions causing distortion of lung architecture. Traction bronchiectasis is typically seen in the within the fibrotic bands.

In the majority of patients with sarcoidosis, sarcoid granulomas resolve over time. However, in approximately 20% of cases, a fibrosing pulmonary pattern may develop [17,55]. The exact pathogenesis of this fibrosing form remains unclear, and it has not been definitively established whether it results from chronic granulomatous inflammation in long-standing disease or represents a distinct disease phenotype [71]. Fibrotic changes in pulmonary sarcoidosis typically include linear opacities, traction bronchiectasis, and architectural distortion, with primary involvement of the upper lobes and peribronchovascular regions [9] (Figure 5). Additionally, “honeycombing” changes can be seen in approximately 10% of cases [72]. The location of these changes, especially in the upper-middle lobes, helps distinguish sarcoidosis from other interstitial lung diseases, which tend to affect the entire lung parenchyma or are more commonly associated with lower lobe involvement [9].

### 5.1. Progressive Fibrosing Form

Although fibrosis in sarcoidosis is generally not progressive, it can evolve in up to 13% of chronic cases [73], albeit at a slower rate than in other interstitial lung diseases.

Predicting the natural course of pulmonary sarcoidosis remains a challenge in clinical practice. As reported by Marll et al., persistent granulomatous inflammation leads to pulmonary fibrosis in sarcoidosis patients [74]. And in this context, there is a clinical need for research on the role of anti-fibrotic therapy for those patients. In this complex clinical setting, there is a need to identify specific features that can predict, from the onset, the risk of progression [75].

In clinical practice, the radiological progression of fibrosis is commonly assessed through semi-quantitative analysis, where visual evaluation of HRCT images is used to estimate the percentage of lung volume affected by fibrosis compared to prior imaging. Although more advanced quantitative systems utilizing artificial intelligence (AI) software have been developed, these are not yet standardized or widely accessible [19,76,77,78].

Thus, it remains critical for patients with pulmonary sarcoidosis to undergo regular monitoring and timely diagnosis of any radiological progression toward a progressive fibrosing phenotype. This ensures appropriate multidisciplinary management and the timely introduction of anti-fibrotic treatments when necessary [19,79]. The key characteristics of fibrosing sarcoidosis are summarized in Table 3.

### 5.2. Association with Idiopathic Pulmonary Fibrosis

Idiopathic pulmonary fibrosis (IPF) is a chronic, progressive fibrosing lung disease diagnosed in the absence of an identifiable cause, typically affecting older male smokers [80]. The hallmark radiological and histopathological finding in IPF is usual interstitial pneumonia (UIP), characterized by “honeycomb” lung areas, and sometimes mediastinal lymphadenopathy [80].

Despite their differences in etiology, epidemiology, risk factors, symptoms, and imaging features, recent studies have proposed an overlap phenotype between sarcoidosis and IPF, termed CSIPF—Combined Sarcoidosis and Idiopathic Pulmonary Fibrosis. Patients with CSIPF often exhibit more rapid functional decline than those with isolated IPF or fibrotic sarcoidosis. It remains unclear whether CSIPF represents a progression of fibrosing sarcoidosis or constitutes a distinct disease entity [81].

## 6. Airways

Airway involvement in sarcoidosis is an atypical manifestation, often resulting from indirect processes such as extrinsic compression by enlarged lymph nodes or distortion of the airways caused by granulomatous inflammation. These changes can affect both the upper and, more commonly, the lower tracheobronchial tree [9,82].

Involvement of the upper respiratory tract is seen in approximately 5% of cases and can affect the nose, paranasal sinuses, larynx, oral cavity, ears, trachea, and bronchi [83,84]. The initial morphological lesion typically involves mucosal edema, followed by granuloma formation, which can coalesce into the characteristic “cobblestone” pattern [9]. Isolated or coalescent endobronchial granulomas may cause bronchial occlusion, leading to airway obstruction or stenosis, and resulting in air trapping—particularly visible in expiratory-phase imaging—and subsegmental atelectasis.

**Figure 6:**
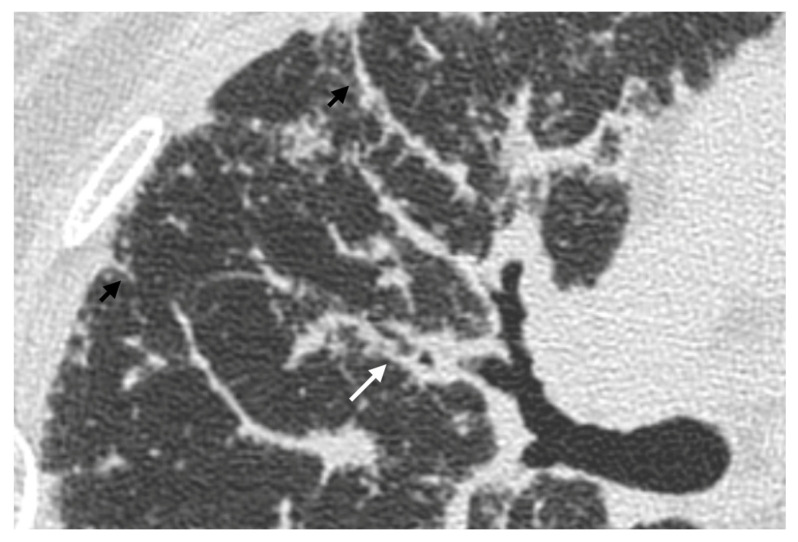
Axial HRCT scan shows diffuse micronodules with a peri lymphatic distribution (black arrows) and a nodular and irregular bronchial wall thickening caused by endobronchial granulomas (withe arrow).

In atypical forms of pulmonary sarcoidosis, tracheobronchial abnormalities can arise from extrinsic compression by parenchymal nodules, enlarged lymph nodes, or less frequently bronchial distortion due to inflammatory changes (Figure 6). These anomalies most often affect the right middle lobe bronchus, which, due to its anatomical and lymphatic characteristics, carries a higher risk of obstruction [85,86,87].

## 7. Pulmonary Hypertension

Pulmonary hypertension (PH) has a multifactorial origin, with extensive interstitial fibrosis in sarcoidosis being a known risk factor [88]. PH is a negative prognostic indicator, associated with a mortality rate seven times higher than in patients without it [82]. It can also result from tracheobronchial abnormalities caused by extrinsic compression from nodules, parenchymal masses, or enlarged lymph nodes [85,87].

The diagnosis of PH is confirmed by a mean pulmonary arterial pressure of at least 20 mmHg, measured through heart catheterization. Imaging findings suggestive of PH include dilation of the main pulmonary artery (>29 mm) [89], dilated right and left pulmonary arteries, right ventricular enlargement, and an increased ratio between the diameter of the main pulmonary artery and the thoracic aorta (>1.1) [17,72].

## 8. Main Differential Diagnoses

### 8.1. Pulmonary Metastases

**Figure 7:**
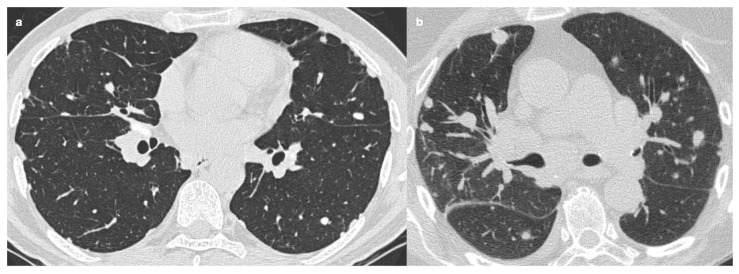
The HRCT scan of a patient with known sarcoidosis shows the presence of diffuse bilateral nodules (**a**). In a patient with known primary malignancy, the lung CT scan shows the presence of metastatic nodules in both lungs of heterogeneous sizes (**b**).

Sarcoidosis can present as a solitary mass or, more commonly, multiple well-defined, rounded nodules with axial diameters greater than 5 mm, which may resemble a solitary pulmonary nodule or metastatic lesions [2]. One of the most challenging aspects of diagnosis is distinguishing sarcoidosis from metastases on imaging. It has been suggested that a granulomatous reaction to a neoplasm is typically localized to the neoplasm’s vicinity [90], making it essential to investigate other potential granulomas in different sites. In more complex cases, particularly in oncological patients, histological analysis may be required to confirm the diagnosis [72] (Figure 7).

Certain neoplasms, such as lymphoma and small-cell carcinoma, can also present with bilateral hilar lymphadenopathy. However, in the absence of specific symptoms or clinical signs, sarcoidosis should remain the primary consideration.

Table 4 summarizes the key differentiating features between sarcoidosis and pulmonary metastases.

### 8.2. Carcinomatous Lymphangitis

In rare cases of sarcoidosis, significant and heterogeneous thickening of the interlobular septa may resemble the appearance of carcinomatous lymphangitis.

Smooth reticular opacities are present in approximately 50% of patients with sarcoidosis, and in 15–20% of cases, they represent the predominant radiological pattern. These opacities result from thickening of the interlobular and intralobular septa, most commonly seen in the subpleural regions of the upper and middle lung lobes [61,67,91,92]. In certain cases, marked and irregular interlobular septal thickening may mimic the radiological appearance of carcinomatous lymphangitis, necessitating careful differential diagnosis [93].

Table 5 summarizes the main differentiating features between sarcoidosis and carcinomatous lymphangitis.

### 8.3. Tuberculosis

Tuberculosis (TB) is an infectious disease primarily caused by *Mycobacterium tuberculosis*, which predominantly affects pulmonary parenchyma. A distinctive feature of lymphadenopathy in sarcoidosis is its asymmetrical distribution [53]. In contrast, unilateral calcified hilar lymphadenopathy is more suggestive of a past TB infection (61%) than of sarcoidosis (23%) [72].

**Figure 8:**
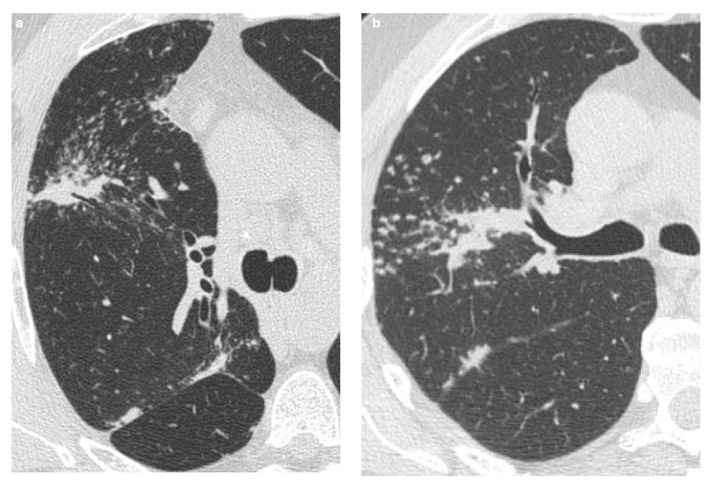
An example of “galaxy sign” in a patient with sarcoidosis (**a**) and in a patient with Tuberculosis (**b**).

Radiological findings in TB can overlap with those in sarcoidosis, including the “galaxy sign” (i.e., central nodule surrounded by hazy or ground-glass opacity) [53,94] (Figure 8), the “sarcoid mass sign” (i.e., small, non-conglomerated granulomas appearing as masses) [95], and the “reverse halo sign” (or “atoll sign”, i.e., ring-shaped consolidation with a central area of ground-glass opacity, usually associated with active inflammation) [68,69,96]. In cases of uncertainty, a history of potential exposure to infectious agents, combined with targeted laboratory tests, can be fundamental for distinguishing between the two conditions [53].

Table 6 summarizes the main differentiating features between sarcoidosis and TB.

### 8.4. Organizing Pneumonia

Organizing pneumonia (OP) shares some radiological features with sarcoidosis, such as areas of lung consolidation that may be associated with ground-glass opacities, often preferentially distributed in the subpleural and bronchovascular regions on both sides [10]. In some cases, the “reverse halo sign” may also be observed [55,97,98].

However, there are distinguishing features between OP and sarcoidosis. Specifically, OP may demonstrate migration or spontaneous resolution of the parenchymal consolidations, a finding less common in sarcoidosis. Additionally, pleural effusion is more frequently seen in OP, occurring in up to 10–35% of cases [98,99].

### 8.5. Silicosis

**Figure 9:**
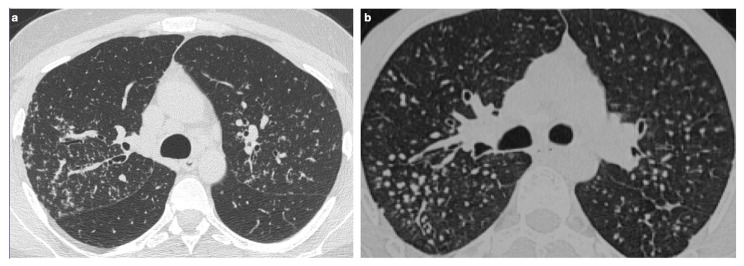
An example of typical sarcoidosis characterized by the presence of diffuse micronodules with perilymphatic distribution (**a**). The HRCT of a patient with occupational exposure to silicium is characterized by the presence of diffuse nodules predominantly in the upper lobes (**b**).

Silicosis can present with radiological findings similar to those seen in sarcoidosis, including micronodules predominantly in the upper lung lobes and enlarged, sometimes coalescent (up to 4 cm), hilar and mediastinal lymph nodes with “egg-shell” calcifications [2,100] (Figure 9). A distinguishing feature that may favor a diagnosis of sarcoidosis is the bilateral nature of these calcifications [72].

Additionally, in sarcoidosis, conglomerate opacities generally originate from the hilar region and extend posteriorly, with or without reticulations, traction bronchiectasis, and architectural distortion. In contrast, in silicosis, conglomerate opacities typically arise from the perilobar region, may affect the upper lobes, and can undergo calcification or cavitation. Dense fibrotic areas in silicosis can also extend to the peripheral lung parenchyma [2].

In some cases, a thorough patient history, particularly relating to professional or silica dust exposure, may be crucial for distinguishing between the two conditions [101].

Table 7 summarizes the main differentiating features between sarcoidosis and silicosis.

### 8.6. Common Variable Immunodeficiency

Common Variable Immunodeficiency (CVID) is a primary immunodeficiency disorder characterized by low serum levels of immunoglobulins and B-cell dysfunction [102,103]. In some cases, CVID presents as a granulomatous and lymphoproliferative disease, referred to as granulomatous-lymphocytic interstitial lung disease (GLILD). This condition primarily affects the small airways and pulmonary interstitium, making it difficult to distinguish from sarcoidosis [3,102,104].

On CT imaging, CVID is typically characterized by poorly defined nodules, which may be distributed randomly or in a centrilobular fashion, with greater involvement in the middle-lower lobes. However, there have been reports where well-defined nodules adopt a perilymphatic distribution with hilar and mediastinal lymphadenopathy, thus resembling sarcoidosis more closely [3,102,105].

Both diseases can present with non-necrotizing granulomas. However, features suggestive of CVID include the presence of organized pneumonia and follicular bronchiolitis (which are rarely seen in sarcoidosis), low serum immunoglobulin levels, and a history of recurrent infections [3,102,105].

Table 8 compares the main features of sarcoidosis and CVID.

## 9. Sarcoidosis and Neoplasms

### 9.1. Possible Correlations Between Neoplasms and Concurrent Onset of Sarcoidosis

The association between neoplastic diseases and the concurrent onset of sarcoidosis remains a subject of debate, with studies showing conflicting results. These discrepancies often arise from the heterogeneity of the study populations and the focus on various types of tumors [106,107,108], without accounting for the immunosuppressive effects of ongoing cancer therapies [109].

Despite these challenges, the two conditions can coexist [110]. However, differentiating sarcoidosis from granulomatous reactions associated with neoplasms is not always straightforward, as both can exhibit similar histological features. PET-CT with 18-FDG can assist in the diagnosis, but the biopsy remains the gold standard to differentiate between reactive lymph nodes and neoplastic ones [111,112].

The simultaneous onset of sarcoidosis and neoplasms may represent an immune response to tumors, commonly referred to as “sarcoid-like reactions” (SLRs). These granulomatous manifestations are thought to occur as a result of immune system activation, in response to tumor proliferation or immunostimulatory oncological treatments (e.g., PD-1/PD-L1 inhibitors). This relationship suggests an intrinsic immune mechanism, with sarcoidosis potentially serving an ambivalent role: acting as a natural immune barrier against metastasis but, over time, leading to immune overload and persistent macrophage activation, which can contribute to the development of hematological neoplasms like lymphoma, monoclonal gammopathy of undetermined significance (MGUS), and macroglobulinemia [109,113,114].

### 9.2. Drug-Induced Sarcoidosis-like Reactions

**Figure 10:**
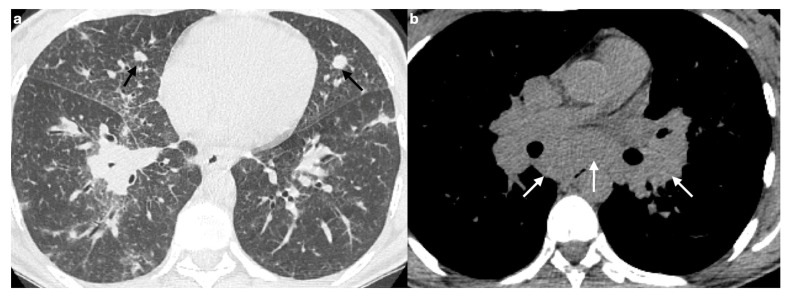
Sarcoidosis-like reaction is indistinguishable from sarcoidosis and is related to initiation of an offending drug. (**a**) CT scan shows bilateral parenchymal nodules (black arrows) and (**b**) the enlargement of hilar and mediastinal lymph nodes (white arrows) after the initiation of TNF-*α* antagonists.

Sarcoid granulomas result from a complex interplay between innate and adaptive immune responses in genetically predisposed individuals, and the distribution of nodules reflects the pathophysiology of the disease. However, it is important to recognize that significant overlap exists with other conditions [100]. Particularly relevant is the drug-induced sarcoidosis-like reaction (DISR), where certain medications can induce a systemic granulomatous syndrome clinically indistinguishable from sarcoidosis (Figure 10). DISR often shows a close temporal relationship to the initiation of the implicated drug, and discontinuing the therapy can lead to improvement or even complete resolution of the condition [1]. The resolution of symptoms after drug discontinuation and recurrence upon re-administration are key indicators that distinguish DISR from sarcoidosis.

The main categories of drugs implicated in DISR include: combination antiretroviral therapies (cART), tumor necrosis factor-*alpha* (TNF-*α*) antagonists, interferon (IFN)-based drugs, and immune checkpoint inhibitors (ICIs) [115].

## 10. Rare Complications of Pulmonary Sarcoidosis

### 10.1. Cavitation

Cavitary lesions may be present in approximately 10% of patients with sarcoidosis, with primary cavitary sarcoidosis occurring in around 2% of cases, typically in those with more severe forms of the disease. In these cases, it is crucial to assess the possibility of concurrent fungal or mycobacterial infections [18,20,56].

### 10.2. Fungal Colonization

Fungal infections, particularly *Aspergillus*, complicate 3% to 12% of sarcoidosis cases with cavitary or bullous pathology. Aspergillus infection typically presents as a simple aspergilloma, especially in the upper lung lobes, within a densely fibrotic lung, a pre-existing emphysematous bulla, or an ectatic airway [20,116,117,118]. An aspergilloma consists of saprophytic fungal elements, fibrin, mucus, and cellular debris, encased by epithelium but lacking pericavitary fibrosis, in contrast to complex aspergillomas, which do exhibit fibrosis. On a CXR, aspergillomas are seen as rounded masses surrounded by a radiolucent semi-circle, which separates the mass from the cavity wall, a feature known as the “Monod sign” or “semicircle sign” [53].

In a small percentage of patients, inadequate treatment of fungal colonization can lead to chronic and extensive fibrotic destruction [20,117]. However, death typically results from advanced pulmonary sarcoidosis rather than from hemoptysis caused by aspergillomas [4,118].

### 10.3. Pleural Pathologies

Pleural thickening is relatively rare in sarcoidosis but may still occur. It is thought to be mimicked by inward retraction of the pleura and soft extra-thoracic tissues, particularly in the fibrotic form of pulmonary sarcoidosis, contributing to restrictive pulmonary dysfunction. Even less frequently, pleural effusion and pneumothorax have been reported, with pneumothorax generally occurring only in the presence of bullous disease [20,119,120,121,122,123].

## 11. Conclusions

Pulmonary sarcoidosis is highly variable and can present with a broad spectrum of imaging patterns. This diversity of radiological findings means that sarcoidosis can mimic a wide range of other diseases, earning it the nickname “the great pretender”.

It is essential for radiologists to be familiar with both the typical and atypical manifestations of sarcoidosis to promptly identify signs that could point to other conditions and make a precise differential diagnosis—particularly when distinguishing it from tuberculosis, silicosis, and lymphoma.

Ultimately, radiologists play a pivotal role in ensuring an accurate diagnosis and recognizing complications, which is critical for the effective management of patients with sarcoidosis.

## Figures and Tables

**Table 1:** Siltzbach classification system for pulmonary sarcoidosis.

Stage	Description
Stage 0	Normal chest radiograph, no evidence of disease.
Stage I	Bilateral hilar lymphadenopathy, often associated with enlargement of right para-tracheal lymph nodes, without parenchymal lung alterations.
Stage II	Bilateral hilar lymphadenopathy accompanied by reticular parenchymal opacities.
Stage III	Reticular parenchymal opacities without hilar lymphadenopathy.
Stage IV	Reticular opacities associated with pulmonary volume loss, predominantly involving the upper lobes. Possible presence of traction bronchiectasis, calcifications, cavitations, or cystic formations.

**Table 2:** Characteristics of sarcoid granulomas in pulmonary imaging.

Characteristic	Description
Distribution	Perilympathic (75–90% of cases); bilateral and symmetric involvement, especially in the middle and upper lobes
Nodule size	Micronodules 2–4 mm in diameter
Morphology	Nodules with well-defined margins, round shape
HRCT localization	Peribronchovascular interstitium, interlobar fissures, interlobular septa
Evolution	Possible coalescence of micronodules into macronodules over time

**Table 3:** Characteristics of fibrosing sarcoidosis.

Characteristic	Description
Incidence	Approximately 20% of patients with sarcoidosis
Origin	Chronic inflammation, long-lasting disease, or phenotypic susceptibility
Alterations	Linear opacities, traction bronchiectasis, architectural distortion
Distribution	Predominantly in the upper lobes and peribronchovascular regions
Honeycombing	Present in about 10% of patients, typically localized in middle-upper lobes

**Table 4:** Main features of sarcoidosis vs. pulmonary metastases.

Characteristic	Sarcoidosis	Pulmonary Metastases
Presence of nodules or masses	Rarely presents a single mass or solitary nodule	Frequently presents multiple well-defined, rounded nodules
Appearance of nodules	May have macronodules (>5 mm), multiple, well-defined, simulating metastases	Multiple, well-circumscribed nodules distributed across various lobes
Differential diagnosis	Difficult to distinguish from metastatic disease with imaging alone	Often requires exclusion of granulomatous diseases
Granuloma localization	Granulomas may appear near tumors, but also in distant sites	Metastatic nodules develop from a known or unknown primary tumor
Diagnostic strategy	Search for granulomas in other sites and confirm via biopsy/histology in doubtful cases	Diagnosis based on oncological history and histopathological confirmation, if necessary

**Table 5:** Main features of sarcoidosis vs. carcinomatous lymphangitis.

Characteristic	Sarcoidosis	Carcinomatous Lymphangitis
Interlobular septa thickening	Marked	Moderate or absent
Involvement of subpleural interstitium	Extensive	Limited
Distribution	Often asymmetric, irregular	Symmetric, bilateral
Peribronchovascular nodules	Rare	Frequent (perilymphatic distribution)
Nodules > 1 cm	Rare	Infrequent
Overall appearance	Infiltrative, along the lymphatic vessels	Nodular, well-defined

**Table 6:** Main features of sarcoidosis vs. tuberculosis.

Characteristic	Sarcoidosis	Tuberculosis
Etiological agent	Non-infectious, immunologic	Infectious (mainly *Mycobacterium tuberculosis*)
Lung parenchyma	Frequently affected	Frequently affected
Galaxy sign	Variably present (14–54%)	Rare (<5%)
Sarcoid mass sign	Variably present (10–20%)	Infrequent (5–10%)
Nodular distribution	More regular, mainly along the bronchovascular bundle	Random, especially in miliary TB
Unilateral calcified lymphadenopathy	Less common (23%)	Common (61%)
Bilateral lymph node calcifications	Common (65%)	Rare (8%)
“Reverse halo” (or “atoll”) sign	May be present (5–10%)	May be present (<5%)
Differential diagnosis	Requires exclusion of infection	Requires infectious history and specific tests (active vs. latent TB)

**Table 7:** Main features of sarcoidosis vs. silicosis.

Characteristic	Sarcoidosis	Silicosis
Type of opacity	Perilobar conglomerate opacities	Perilobar conglomerate opacities, possible calcifications and cavitations
Origin and extension	From the hilar region, extending posteriorly	Fibrotic areas with bands extending to the periphery
Other radiological signs	Reticulation, traction bronchiectasis, architectural distortion	Dense fibrosis
Preferred location	Middle-upper regions	Upper and perilobar regions
History	Not specifically occupational	Occupational exposure (e.g., silica)

**Table 8:** Main features of sarcoidosis vs. CVID.

Characteristic	Sarcoidosis	CVID
Type of disease	Granulomatous inflammation of unknown origin	Primary immunodeficiency with granulomatous and lymphoproliferative manifestations
Immunoglobulins levels	Normal	Reduced (e.g., hypogammaglobulinemia)
History	Generally, absence of recurrent infections	Presence of recurrent bacterial infections
Typical CT appearance	Perilymphatic nodules, hilar and mediastinal lymphadenopathy	Poorly defined nodules, centrilobular or random distribution
Atypical/overlapping CT appearance	Macronodules, sometimes coalescent, and unilateral hilar lymphadenopathy	Possible perilymphatic nodules, with lymphadenopathy similar to sarcoidosis
Pulmonary involvement	Common, with perilymphatic distribution	Common, with involvement of small airways and interstitium (GLILD)
Granulomas	Non-necrotizing	Non-necrotizing
Distinctive features	Absence of organized pneumonia or follicular bronchiolitis	Presence of organized pneumonia and follicular bronchiolitis
Diagnosis	Biopsy, clinical and radiological context	Biopsy, immunohistochemical tests (i.e., CD3, CD4, CD8, CD20) and clonality evaluation

## Data Availability

No new data were created or analyzed in this study. Data sharing is not applicable to this article.

## Abbreviations

The following abbreviations are used in this manuscript:

GLD	Granulomatous lung disease
CXR	Chest radiography
HRCT	High-resolution computed tomography
18-FDG PET/CT	Positron-emission tomography with fluorodeoxyglucose
MRI	Magnetic resonance imaging
BAL	Bronchoalveolar lavage
EBUS-TBNA	Endobronchial ultrasound-guided transbronchial needle aspiration
PFT	Pulmonary function test
FVC	Forced vital capacity
FEV1	Forced expiratory volume in one second
DLCO	Diffusing capacity of the lung for carbon monoxide
6MWT	Six-minute walking test
GGO	Ground-glass opacities
IPF	Idiopathic pulmonary fibrosis
CSIPF	Combined sarcoidosis and idiopathic pulmonary fibrosis
PH	Pulmonary hypertension
TB	Tuberculosis
OP	Organizing pneumonia
CVID	Common variable immunodeficiency
GLILD	Granulomatous-lymphocytic interstitial lung disease
SLR	Sarcoid-like reaction
MGUS	Monoclonal gammopathy of undetermined significance
DISR	Drug-induced sarcoidosis-like reaction
cART	Combination antiretroviral therapy
TNF-*α*	Tumor necrosis factor-*alpha*
IFN	Interferon
ICI	Immune checkpoint inhibitor
